# Improvement of nerve imaging speed with coherent anti-Stokes Raman scattering rigid endoscope using deep-learning noise reduction

**DOI:** 10.1038/s41598-020-72241-x

**Published:** 2020-09-16

**Authors:** Naoki Yamato, Hirohiko Niioka, Jun Miyake, Mamoru Hashimoto

**Affiliations:** 1 Graduate School, Faculty of Information Science and Technology, Hokkaido University, Kita 14 Nishi 9 Kitaku, Sapporo, 060-0814 Japan; 2 Institute for Datability Science, Osaka University, 2-8 Yamadaoka, Suita, 565-0871 Japan; 3 Hitz Research Alliance Laboratory, Graduate School of Engineering, Osaka University, 2-8 Yamadaoka, Suita, 565-0871 Japan

**Keywords:** Imaging and sensing, Raman spectroscopy, Adaptive optics, Biophotonics, Endoscopy, Medical imaging

## Abstract

A coherent anti-Stokes Raman scattering (CARS) rigid endoscope was developed to visualize peripheral nerves without labeling for nerve-sparing endoscopic surgery. The developed CARS endoscope had a problem with low imaging speed, i.e. low imaging rate. In this study, we demonstrate that noise reduction with deep learning boosts the nerve imaging speed with CARS endoscopy. We employ fine-tuning and ensemble learning and compare deep learning models with three different architectures. In the fine-tuning strategy, deep learning models are pre-trained with CARS microscopy nerve images and retrained with CARS endoscopy nerve images to compensate for the small dataset of CARS endoscopy images. We propose using the equivalent imaging rate (EIR) as a new evaluation metric for quantitatively and directly assessing the imaging rate improvement by deep learning models. The highest EIR of the deep learning model was 7.0 images/min, which was 5 times higher than that of the raw endoscopic image of 1.4 images/min. We believe that the improvement of the nerve imaging speed will open up the possibility of reducing postoperative dysfunction by intraoperative nerve identification.

## Introduction

Visualization of nerves is one of the problems to be solved in nerve-sparing surgery. Since injury to nerves causes serious dysfunction^1^, surgeons should preserve the nerves as much as possible while resecting lesions of the tumor completely. Relatively thick nerves can be identified by eye using the anatomical knowledge possessed by surgeons, but the identification of thin nerves is difficult. Visualization and identification of nerves have been investigated using a number of methods, including electrical stimulation^2,3^, fluorescent peptides^4,5^, and Raman microscopy^6,7^. Electrical stimulation can be applied only to motor nerves since it involves detection of their mechanical response and is not an imaging method per se. Obtaining approval for new fluorescent dyes for labeling healthy nerves is a long and arduous task. One alternative approach to label-free imaging is Raman microscopy; however, nerve imaging with conventional spontaneous Raman scattering requires a long exposure time because of the considerably low efficiency of Raman scattering. Therefore, a label-free, high-imaging-rate method is desired for intraoperative nerve visualization.

We have developed a coherent anti-Stokes Raman scattering (CARS) rigid endoscope for visualizing myelinated nerves without labeling^8,9^. CRS (coherent Raman scattering) imaging, including CARS imaging, provides chemical images based on molecular vibrations, similar to spontaneous Raman scattering microscopy^10–12^. Nerve identification was achieved by visualization of lipids in myelinated nerves using the $\mathrm {CH_2}$ symmetric stretching vibration at 2845 $\mathrm{cm^{-1}}$^13,14^. We achieved the visualization of rat sciatic nerves and rabbit periprostatic nerves with the developed CARS endoscope. Even though we successfully performed CARS imaging of sciatic nerves at an imaging rate of 6 images/min, it required only 1 images/min for more realistic imaging of low-density peripheral nerves in prostate tissues. The reason for the slow imaging rate is that the long exposure time is required to obtain images with a sufficient signal-to-noise ratio. The intensity of the CRS signal is dependent on the numerical aperture (NA) of the objective lens. Using a high NA objective lens in CRS microscopy, typically $>1.0$, has accomplished imaging at rates faster than video rates^15,16^. The previously reported CRS endoscopy systems used large NA and provided the field of view less than 380 ${\upmu \mathrm {m}}$^14,17,18^. The low NA (0.26) of our CARS endoscope, which was used to increase the field of view to 650 ${\upmu \mathrm {m}}$, limited the imaging rate.

Here, we apply deep learning to denoising in order to improve the nerve imaging rate with the developed CARS rigid endoscope. Deep learning has attracted attention in many fields along with the technological progress of Graphics Processing Units (GPUs). Deep learning outperforms conventional methods in image processing, such as image recognition^19–21^, semantic segmentation^22,23^, and denoising^24–26^. Obtaining an image of sufficient quality by denoising of images observed with a high imaging rate (short exposure time) increases the imaging rate. We employ a denoising technique using deep learning for endoscopic nerve imaging. We propose a new evaluation metric called the equivalent imaging rate (EIR), and use the EIR to quantitatively evaluate the improvement of the imaging rate.

**Figure 1:**
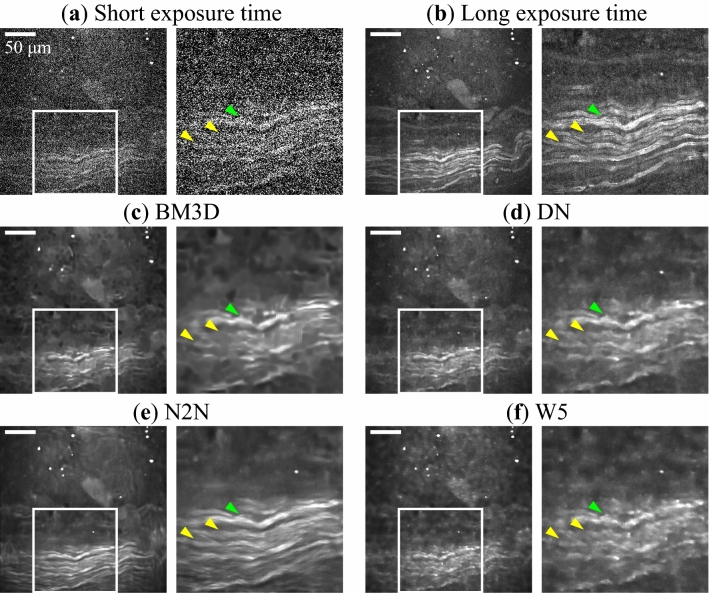
Denoising results of CARS microscopy images. The images are for: **(a)** input observed with an imaging rate of 33.3 images/min (a short exposure time of 1.8 s), **(b)** ground truth observed with an imaging rate of 0.7 images/min (a long exposure time of 90 s), and images denoised with **(c)** the BM3D filter, **(d)** DN, **(e)** N2N, and **(f)** W5. Parts **(a–f)** are each composed of two images: the right image is a magnified image of the white rectangle in the left image. The image size is $270 \times 270\, \upmu \hbox {m}$, with $500 \times 500$ pixels. Nerves lie horizontally in the lower half of the images. Scale bar indicates $50\, \upmu \hbox {m}$. The polarization direction of two laser beams is horizontal in images.

**Table 1:** Comparison of denoising performance between BM3D filter and deep learning models.

Model	Evaluation metrics	
	PSNR	SSIM
BM3D	$31.04 \pm 5.86$	$0.649 \pm 0.207$
DN	$32.05 \pm 6.02$	$0.671 \pm 0.220$
N2N	$\mathbf {32.21 \pm 6.04}$	$\mathbf {0.679 \pm 0.214}$
W5	$31.96 \pm 6.02$	$0.668 \pm 0.221$

Paired t-test	P-value	
	PSNR	SSIM
N2N vs BM3D	0.045*	0.016*
N2N vs DN	0.0059*	0.027*
N2N vs W5	0.00035*	0.016*

The average and standard deviation of each metric over five test images are shown. The N2N shows the significant difference from the others in a paired t-test ($n=5$, $\mathrm {P} < 0.05$) using the Bonferroni–Holm correction. N2N showed the highest performance for both metrics. * means significant difference after the Bonferroni–Holm correction ($\alpha < 0.05$).

**Figure 2:**
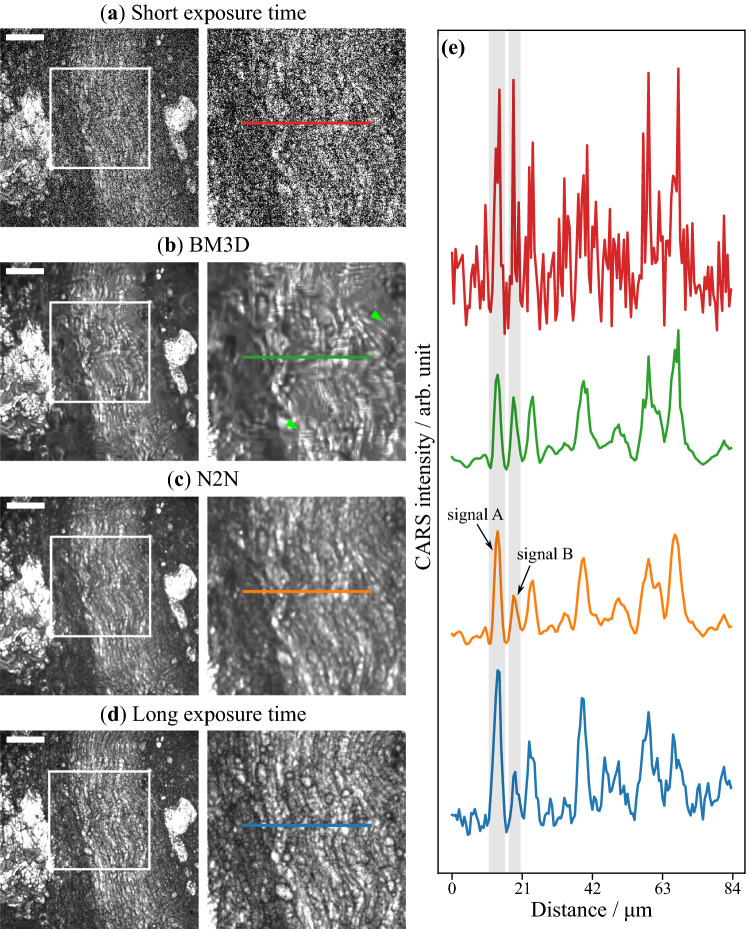
Results of denoised CARS endoscopy images. The images are for **(a)** an input observed with an imaging rate of 12.5 images/min (a short exposure time of 4.8 s), denoised with **(b)** the BM3D filter and **(c)** N2N with ensemble, and **(d)** ground truth observed with an imaging rate of 0.4 images/min (a long exposure time of 160 s). Nerves lie at the center of the images with lipid rich tissue on each side. The CARS images in the second column are images cropped from the white rectangles in the left images. The line profiles of the cropped images are shown in **(e)**. Scale bar indicates $50\, \upmu \hbox {m}$. The polarization direction of two laser beams is horizontal in images.

**Table 2:** Results of denoising CARS endoscopy images.

Model	Evaluation metrics	
	PSNR	SSIM
BM3D	$28.98 \pm 1.12$	$0.690 \pm 0.060$
DN with microscopy	$30.15 \pm 1.73$	$0.690 \pm 0.069$
DN with endoscopy	$30.41 \pm 1.50$	$0.711 \pm 0.052$
DN with fine-tuning	$30.53 \pm 1.54$	$0.713 \pm 0.053$
DN with ensemble	$\mathbf {30.58 \pm 1.69}$	$0.715 \pm 0.058$
N2N with microscopy	$29.97 \pm 1.64$	$0.680 \pm 0.070$
N2N with endoscopy	$30.33 \pm 1.50$	$0.710 \pm 0.055$
N2N with fine-tuning	$30.51 \pm 1.53$	$0.718 \pm 0.054$
N2N with ensemble	$30.56 \pm 1.67$	$\mathbf {0.720 \pm 0.059}$
W5 with microscopy	$30.31 \pm 1.75$	$0.698 \pm 0.066$
W5 with endoscopy	$30.41 \pm 1.51$	$0.712 \pm 0.0520$
W5 with fine-tuning	$30.47 \pm 1.52$	$0.714 \pm 0.052$
W5 with ensemble	$30.50 \pm 1.66$	$0.714 \pm 0.057$

Paired t-test	P-value	
	PSNR	SSIM
DN		
endoscopy vs fine-tuning	0.020	0.15
fine-tuning vs ensemble	0.00070*	0.0072*
N2N		
endoscopy vs fine-tuning	0.0017*	0.0040*
fine-tuning vs ensemble	0.00090*	0.0067*
W5		
endoscopy vs fine-tuning	0.023	0.0087*
fine-tuning vs ensemble	0.014	0.000089*
Comparison of ensemble models		
DN vs N2N	0.52	0.033
DN vs W5	0.029	0.83
N2N vs W5	0.064	0.048
BM3D vs with ensemble		
BM3D vs DN	0.0032*	0.00014*
BM3D vs N2N	0.0030*	0.0000019*
BM3D vs W5	0.0032*	0.00029*

The CARS endoscopy images for input are at 12.5 images/min (the exposure time of 4.8 s). The performances of four learning styles were compared: with microscopy, with endoscopy, with fine-tuning, and with ensemble. The evaluation metrics of the models with fine-tuning were statistically significantly higher than that of the models with endoscopy, except for DN and PSNR of W5. The evaluation metrics of the models with ensemble were statistically significantly higher than that of the models with fine-tuning, except for PSNR of W5. The comparison between the models with ensemble showed no-significant difference, despite having even though P values were < 0.05. The models with microscopy had worse performance than the models with endoscopy in DN and N2N. All of the deep learning models with ensemble showed statistically significantly higher performance than BM3D. * means significant difference by the Bonferroni–Holm correction in paired t-test ($n=5$, $\hbox {P} < 0.05$).

## Results

### Pre-training with CARS microscopy images

We employed a fine-tuning strategy for deep learning models; the fine-tuning was used to retrain pre-trained models. Training of a deep learning model requires a large dataset. In general, 8,000–500,000 images are usually used for training^24,26,27^. However, we could prepare only a small dataset of CARS endoscopy nerve images because of the slow imaging speed. Since the high NA of a CARS microscopy system increases the imaging rate and facilitates nerve exploration, CARS microscopy can easily provide a large dataset of nerve images compared with CARS endoscopy. Therefore, we pre-trained the deep learning models with CARS microscopy images before fine-tuning with CARS endoscopy images. The different fasciae were used in CARS microscopic and endoscopic imaging.

We chose the following three denoising deep learning architectures: DenoiseNet (DN)^28^, Noise2Noise (N2N)^27^, and WIN5R (W5)^29^. DN has a deep convolutional neural network (CNN) architecture and was originally proposed for removing Poisson noise, i.e., signal-dependent shot noise. N2N is based on the U-Net architecture^22^, which is usually applied to semantic segmentation. N2N does not require clean images with a high signal-to-noise ratio for training. W5 is a known shallow CNN architecture for removing signal-independent Gaussian noise. As a classical filter, we selected BM3D^30^, which is known to be one of the most powerful imaging filters. We evaluated and compared the filter and three deep learning models with different architectures.

Figure 1 shows the denoising results of CARS microscopy images obtained with the deep learning models and the filter. Figure 1a,b are images observed with an imaging rate of 33.3 images/min (a short exposure time of 1.8 s) for the input and with an imaging rate of 0.7 images/min (a long exposure time of 90 s) for the ground truth. Figure 1c–f are denoised images obtained with BM3D, DN, N2N, and W5, respectively. Each part of Fig. 1 consists of two images: the right image is a magnified image of the white rectangle in the left image. Figure 1b clearly show the fibrous shape of nerves. In the denoised images shown in Fig. 1c–f, impulse noise shown in Fig. 1a was well removed. The upper nerve fiber bundle indicated by the green arrowheads was well restored and recognized as one bundle. On the other hand, in Fig. 1c,d,f, the nerve fibers indicated by the yellow arrowheads appeared to be broken in the middle. In the N2N case shown in Fig. 1e, the structure of nerve fibers remained. In Fig. 1b, the edge of each nerve fiber shows stronger signals comparing with the central region. This reflects the structure of the lipid-rich myelin sheath covering the axon. However, such fine structures of the myelin sheath and axon were not reconstructed because the lateral resolution was blurring by denoising (Fig. 1c–f).

We used two evaluation metrics to assess the performance of denoising: the peak signal-to-noise ratio (PSNR) and structural similarity (SSIM)^31^. The higher the quality of denoised images, the higher the PSNR and SSIM values. Table 1 shows the averages and standard deviations of these two metrics over five test images. Friedman test ($\mathrm {P} < 0.05$) on 4 models indicated that one of the differences was significant ($\mathrm {P} = 0.0018$ for PSNR and SSIM, respectively). All deep learning models outperformed the BM3D filter. Among deep learning models, N2N showed the highest denoising performance with both metrics. Even though the standard deviations were relatively large because of the high variability of the test images, the paired t-test ($n=5$, $\mathrm {P} < 0.05$) indicated that the two metrics in the N2N case were significantly higher than in the DN case (PSNR: $\mathrm {P} = 0.0059$, SSIM: $\mathrm {P} = 0.027$) and the W5 case (PSNR: $\mathrm {P} = 3.5\times 10^{-4}$, SSIM: $\mathrm {P} = 0.016$) using the Bonferroni-Holm correction.

### Fine-tuning and ensemble learning with CARS endoscopy images

Deep learning models pre-trained with CARS microscopy images were retrained with CARS endoscopy images to enhance the denoising performance for CARS endoscopy images. In addition to fine-tuning, ensemble learning was also applied to denoising. Ensemble learning is a method using multiple models with different weights to achieve better performance compared with a single model. As the ensemble learning models, we used outputs that were the average of four fine-tuned models.

Figure 2 presents the denoising results of a nerve image observed with the CARS endoscope. An input image observed with an imaging rate of 12.5 images/min (a short exposure time of 4.8 seconds) shown in Fig. 2a was denoised with the BM3D filter (b) and N2N (c). Figure 2d is an image observed with an imaging rate of 0.4 images/min (a long exposure time of 160 s) for the ground truth. The right image in each figure was cropped from the white box in the left one. Nerve fibers lie in the center of the images, with sparse lipid-rich tissues on each side. It was found that both BM3D and N2N reduced impulse noise in the input image. However, vertical and horizontal stripes with high frequencies appeared around the green arrowheads in the BM3D denoised image in Fig. 2b. Figure 2e shows the intensity profiles along the color lines in Fig. 2a–d. From the line profiles, we can understand that the noise was well removed in Fig. 2b,c. The ratio of the signal A and B areas in Fig. 2e was 1.14 for BM3D, 1.41 for N2N, and 1.37 for the ground truth. Hence, it seems that N2N provides a higher fidelity signal compared with BM3D.

We used nested cross-validation^32^ to accurately evaluate the denoising performance of the models for CARS endoscopy images. The values of the evaluation metrics are summarized in Table 2, where “with microscopy” means models trained with only CARS microscopy images, “with endoscopy” means models trained with only CARS endoscopy images without pre-training, “with fine-tuning” means models fine-tuned after pre-training, and “with ensemble” means models in which fine-tuning and ensemble learning were combined. Friedman test ($\mathrm {P} < 0.05$) on 13 models indicated that one of the differences was significant (PSNR: $\mathrm {P} = 1.5\times 10^{-7}$, SSIM: $\mathrm {P} = 2.4\times 10^{-7}$). The evaluation metrics of the models “with fine-tuning” were significantly higher than those of “with endoscopy”, except for DN and the PSNR of W5R using the Bonferroni-Holm correction (DN: $\mathrm {P} = 0.020$ (PSNR), 0.15 (SSIM); N2N: $\mathrm {P} = 0.0017$ (PSNR), 0.0040 (SSIM); W5: $\mathrm {P} = 0.023$ (PSNR), 0.0087 (SSIM)). The ensemble learning showed significant enhancement of denoising using the Bonferroni-Holm correction (comparison between fine-tuning and ensemble in the same architecture, DN: $\mathrm {P} = 0.00070\, (\mathrm{PSNR}), 0.0072\, (\mathrm{SSIM})$; N2N: $\mathrm {P} = 0.00090$ (PSNR) , 0.0067 (SSIM); W5: $\mathrm {P} = 0.014$ (PSNR), 0.000089 (SSIM)). The comparisons of the models with ensemble learning show no-significant difference using the Bonferroni-Holm correction despite having P values below 0.05 (DN versus N2N: $\mathrm {P} = 0.52$ (PSNR), 0.033 (SSIM); DN versus W5R: $\mathrm {P} = 0.029$ (PSNR), 0.83 (SSIM); N2N versus W5R: $\mathrm {P} = 0.064$ (PSNR), 0.048 (SSIM)).

**Figure 3:**
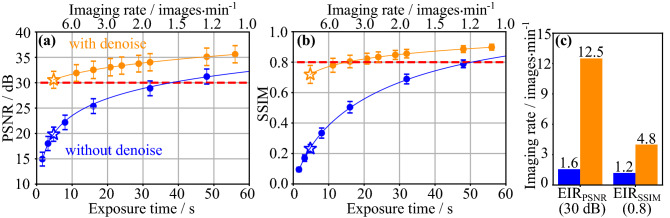
Evaluation of EIR with plots of PSNR **(a)** and SSIM **(b)**. The blue solid circles are the values of evaluation metrics for raw images observed with different imaging rates (exposure times). The orange solid circles are the values of the evaluation metrics for images denoised with N2N with ensemble. The criteria $\mathrm {PSNR} = 30$ dB and $\mathrm {SSIM} = 0.8$ are plotted as the red dashed lines. The star markers in **(a)** and **(b)** correspond the evaluation metrics at 12.5 images/min including Fig. 2c. **(c)** The $\mathrm {EIR_{PSNR}}$ and $\mathrm {EIR_{SSIM}}$ for $\mathrm {PSNR = 30}$ dB and $\mathrm {SSIM = 0.8}$ with (orange) and without denoising (blue), respectively.

We propose the equivalent imaging rate (EIR) as a metric for estimating the improvement of the imaging rate by N2N “with ensemble”. The EIR is defined as a harmonic mean of $\mathrm {EIR_{PSNR}}$ and $\mathrm {EIR_{SSIM}}$, which are imaging rates meeting $\mathrm {PSNR} = 30$ dB and $\mathrm {SSIM} = 0.8$, respectively. In general, $\mathrm {PSNR} > 30$^33^ or $\mathrm {SSIM} > 0.8$^34^ is utilized as a standard criterion indicating that the restored images are indistinguishable from ground truth images. We selected the harmonic mean for averaging the imaging rates of $\mathrm {EIR_{PSNR}}$ and $\mathrm {EIR_{SSIM}}$. The blue plots and fitted curves in Fig. 3a,b show the relationships between the imaging rate and each metric for unprocessed raw CARS endoscopic images. The orange plots and fitted curves are the results for images denoised by using the model N2N “with ensemble” that were trained with the images observed with each imaging rate. The evaluation metrics of the CARS image observed at 12.5 images/min were $19.85 \pm 1.40$ dB (PSNR) and $0.232 \pm 0.027$ (SSIM). These metrics increased to $30.56 \pm 1.67$ dB (PSNR) and $0.720 \pm 0.059$ (SSIM) by noise reduction with deep learning. These results are indicated by star markers in Fig. 3a,b. The large improvements in the evaluation metrics were confirmed for denoising images observed at the high imaging rates. Figure 3c shows the EIRs of the $\mathrm {EIR_{PSNR}}$ and $\mathrm {EIR_{SSIM}}$ of the raw (blue) and denoised (orange) images with the criteria PSNR = 30 dB and SSIM = 0.8. PSNR and SSIM of observed CARS image without denoising satisfied the criteria at 1.6 images/min ($\mathrm {EIR_{PSNR}}$) and 1.2 images/min ($\mathrm {EIR_{SSIM}}$), respectively. By the denoising, the $\mathrm {EIR_{PSNR}}$ and $\mathrm {EIR_{SSIM}}$ increased to 12.5 and 4.8 images/min. As a result, it was found that denoising accelerated EIR, which is the harmonic mean of $\mathrm {EIR_{PSNR}}$ and $\mathrm {EIR_{SSIM}}$, from 1.4 images/min to 7.0 images/min.

**Table 3:** Denoising processing time for one image with BM3D filter and three deep learning architectures.

Model	Averaged time	Parameters
BM3D	$5048 \pm 35$ ms	–
DN	$20.0 \pm 1.9$ ms	0.224 M
DN with ensemble	$79.4 \pm 0.6$ ms	0.897 M
N2N	$15.2 \pm 1.7$ ms	1.342 M
N2N with ensemble	$59.6 \pm 3.0$ ms	5.366 M
W5	$3.3 \pm 0.7$ ms	0.019 M
W5 with ensemble	$12.6 \pm 0.6$ ms	0.075 M

For the deep learning models, the processing times with ensemble learning was measured. The processing time of N2N was faster than that of DN because five pooling layers are combined in N2N. The average and standard deviation from 1000 measurements are shown.

**Figure 4:**
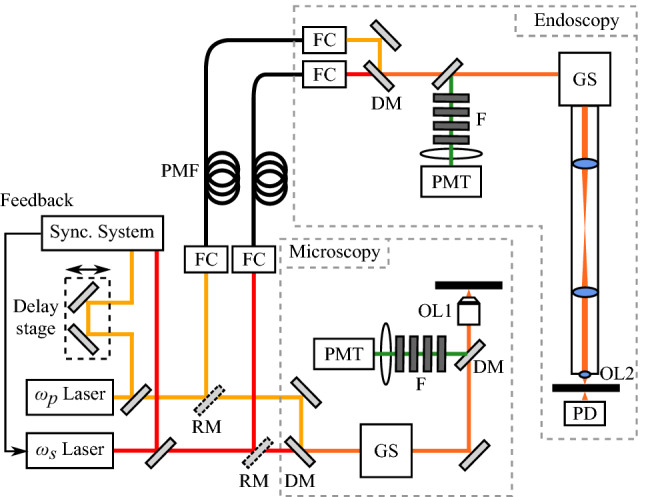
Schematic of the CARS endoscope and microscope system. The two lasers were synchronized with a custom-built electrical device. Each laser beam was transmitted to the endoscopy system via a polarization-maintaining single-mode fiber. The two laser beams were combined with a long pass dichroic mirror and made incident on a galvanometer scanner. The backscattering CARS signal was separated and detected by a photomultiplier tube. The CARS microscope system is similar to the endoscope system, except for the optical fiber. *DM* dichroic mirror, *F* optical filters, *FC* fiber coupler, *GS* galvanometer scanner, *OL* objective lens, *PD* photodiode, *PMF* polarization-maintaining single-mode fiber, *PMT* photomultiplier tube, *RM* removable mirror.

**Figure 5:**
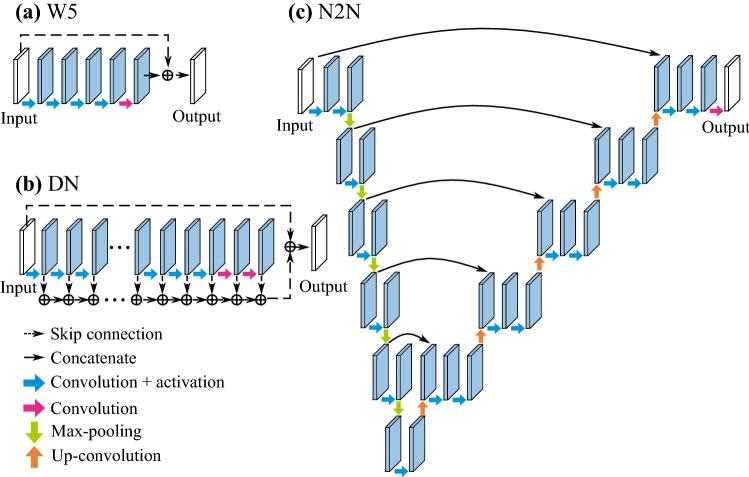
Schematic diagrams of three denoising models.** (a)** The W5 architecture is composed of a skip connection and convolution layers. The convolution layers except for the last layer are followed by a ReLU activation function.** (b)** The DN architecture has a structure similar to W5. The difference is that each convolution layer has a skip connection to the output.** (c)** The N2N architecture is based on U-Net. The convolution layers except for the last layer are followed by a leaky ReLU activation function. The number of filters in the convolution layers of the encoder part is half of the number in the decoder part except for the last three layers.

**Table 4:** Hyperparameters utilized for grid search in this work.

Model	Number of filters	Size of filter	Number of convolution layers
DN	64, 32, 16 or 8	$7\times 7$, $5\times 5$ or $3\times 3$	25,20,15 or 10
N2N	128, 96, 64, 48 or 32	$3\times 3$	18
W5	128, 64, 32, 16 or 8	$7\times 7$, $5\times 5$ or $3\times 3$	10, 8, 6, 5 or 4

## Discussion

We demonstrated that denoising with deep learning improved the nerve imaging rate with CARS endoscopy. Deep learning models for noise reduction were trained with a fine-tuning strategy, in which the models were pre-trained with CARS microscopy images and then retrained with CARS endoscopy images. N2N with ensemble learning showed the highest denoising performance. The EIR of the deep learning model was 7.0 images/min ($\mathrm {EIR_{PSNR}}$: 12.5 images/min, $\mathrm {EIR_{SSIM}}$: 4.0 images/min), whereas those of raw endoscopy imaging were 1.4 images/min ($\mathrm {EIR_{PSNR}}$: 1.6 images/min, $\mathrm {EIR_{SSIM}}$: 1.2 images/min).

We employed the fine-tuning strategy for training of deep learning models using CARS microscopy images as the pre-training dataset. The fine-tuning was significantly effective in most cases by using the same size of acquired images and the same photomultiplier under the same conditions. Therefore, we concluded that it is effective to perform pre-training using images obtained by an easier method that provides similar results, instead of images that are difficult to obtain. Besides, we confirmed how the amount of pre-training images affects on the denoising performance of N2N with fine-tuning (Supplementary Figure S1). Since increasing the pre-training data improved the accuracy of the denoised images, adding more pre-training data will improve the accuracy. For further enhancement of the accuracy, generative adversarial networks (GANs)^35,36^, which can enhance the performance by using a pair of two networks, are strong candidates.

We proposed using the EIR as a new evaluation metric for quantitatively and directly assessing the imaging rate improvement of deep learning models. The EIR is defined as a harmonic mean of $\mathrm {EIR_{PSNR}}$ and $\mathrm {EIR_{SSIM}}$, which mean imaging rates satisfying a certain criterion, namely, $\mathrm {PSNR} = 30$ dB and $\mathrm {SSIM} = 0.8$ here. The PSNR, SSIM, and mean squared error (MSE) are general metrics used to indicate the effect of noise reduction. However, these metrics are independent of the imaging rate, and therefore, we proposed the EIR using these metrics as criteria.

The nerve shape recognition ability of deep learning models seems to be essential for noise reduction in nerve images. W5 is composed of a skip connection and a few convolution layers. In W5, the output image of the last convolution layer is added to the input image. W5 learns that the output of the last convolution layer cancels out the noise in the input image. Since the depth of W5 is small, W5 is considered to have little information about the nerve shape. The architecture of DN is similar to W5, but DN has more skip connections to add the image of each layer to the input image. The outputs of the deeper layers in DN serve to restore specific textures in the input image. N2N consists of an encoder–decoder architecture based on U-Net, which is known to be a model that is superior to shape recognition. The encoder extracts the features of the input image and the decoder reconstructs the output image from the extracted features. We compare the evaluation metrics of the models “with microscopy” and “with endoscopy” in each architecture in Table 2. Although W5 “with microscopy” and “with endoscopy” showed small differences (PSNR: 0.10; SSIM: 0.014), DN showed large differences (PSNR: 0.26, SSIM: 0.021), and N2N also showed large differences (PSNR: 0.36, SSIM: 0.030) in Table 2. The appearance of nerves observed with CARS endoscopy seemed to differ from those observed with CARS microscopy due to the difference of NA between the microscopy system ($\mathrm {NA} = 1.1$) and the endoscopy system ($\mathrm {NA} = 0.26$). In CARS microscopy, sectioned nerve images are obtained by using the high NA objective lens. On the other hand, in CARS endoscopy, nerve fibers appear to overlap due to the low axial resolution by the low NA. We considered that DN and N2N “with microscopy” showed lower evaluation metrics because of the difference in the axial resolution. In other words, DN and N2N learned the nerve shape. We believe that N2N will be able to learn more particular shapes by utilizing its architecture, and will be more effective for observing specific organs.

Implementing a denoising technique with deep learning for medical settings has two advantages. First, denoising with deep learning is faster than that with the conventional filter. The processing time of denoising with each model is summarized in Table 3. The processing time of N2N is shorter than that of DN due to the pooling layers in N2N. When ensemble learning is employed, the four models could be implemented in the form of parallel processing. The processing time with the deep learning model was negligible compared with the exposure time. Second, the biggest advantage of N2N is the possibility of updating the model with retraining during surgery. Fine-tuning of the denoising model is needed when introducing the model for the first time, for infrequent surgery, or when the conditions of the optical system change. It is difficult to prepare adequate data and fine-tune the model at all such times because obtaining the ground truth data for a long time is laborious. A learning style that does not involve observing the ground truth allows for in situ fine-tuning in a situation where an image is taken two times at the same position. The possibility of maintaining the model by fine-tuning is important for surgical use. Currently, off-line processing is being performed, but we will demonstrate the improvement of imaging rate by on-line processing in the future.

A further challenge is nerve segmentation to help surgeons recognize nerves. The present nerve imaging by CARS endoscopy is based on the $\mathrm {CH_2}$ symmetric stretching vibration at 2,845 $\mathrm{cm^{-1}}$. CARS endoscopy images include signals of lipid-rich tissues in addition to nerves, making it difficult to recognize the nerves. Semantic segmentation, which classifies objects at each pixel in an image, may be useful for extracting and displaying the nerve area. Semantic segmentation with deep learning performs significantly better than classical methods and can be used in conjunction with deep learning denoising. Therefore, a combination of semantic segmentation and CARS endoscopy imaging will contribute to the realization of intraoperative nerve imaging. In addition to image processing, the introduction of endoscopic surgery requires expanding the field of view and improving the portability of the CARS endoscopy system. Our CARS endoscope has a wider field of view comparing other CARS (CSR) endoscopes. Expanding the field of view will improve nerve discernibility. Also replacing the solid-state lasers with fiber lasers^37^ will improve the portability and handling of the CARS endoscope. The CARS endoscope will identify the thin nerve and reduce the dysfunction after surgery.

## Methods

### Sample preparation

Rabbit urinary organs including prostates and peri-prostatic fasciae were purchased from the Japan Lamb Co., Ltd., and were preserved in 4% paraformaldehyde phosphate buffer solution. The formalin fixation does not deteriorate the CARS signal of lipid component in tissue and cell samples^38,39^. The peri-prostatic fasciae were excised from the rabbit urinary organs, rinsed with PBS, and sandwiched between two cover slips within PBS to prevent drying of the fasciae.

### CARS microscopy

A picosecond mode-locked Ti:sapphire laser (Tsunami, Spectra-Physics; repetition rate $= 80$ MHz) and a picosecond acoustic-optic tunable filter (AOTF) mode-locked Ti:sapphire laser (Megaopt; repetition rate $= 80$ MHz) were used as excitation light sources, as shown in Fig. 4, serving as $\omega _p$ and $\omega _s$ laser beams, respectively. The synchronization system of the two lasers was previously reported^40,41^. The wavelengths of these lasers were tuned to 709 nm and 888 nm to excite the $\mathrm {CH_2}$ symmetric stretching vibration of lipids at 2845 cm$^{-1}$. Two laser beams were superimposed at a long-wavelength-pass filter and were focused with an objective lens (CFI Apo LWD, $\times 25$, $\mathrm {NA} = 1.1$, WI, Nikon). Backward-propagating CARS was collected with the same objective lens and was detected with a photomultiplier tube (PMT; R9110, Hamamatsu). The powers of the two excitation beams on the sample plane were 31 mW for 709 nm and 11 mW for 888 nm. We observed 100 nerve images (field of view of $273 \,\upmu \mathrm {m}$, $500 \times 500$ pixels, imaging rate: 66.7 images/min, exposure time: 0.9 s) at one position with CARS microscopy, and prepared a total of 9100 nerve images at 91 positions. An average image of 100 images at one position was used as a ground truth image.

### CARS endoscopy

The CARS endoscopy system was previously reported^9^. The rigid endoscope has a length of 270 mm and a diameter of 12 mm. The same laser system and wavelengths as with the CARS microscopy described above were used as excitation light, as shown in Fig. 4, to serve as $\omega _p$ and $\omega _s$ laser beams, respectively. The two beams were individually delivered to the endoscope head via polarization-maintaining single-mode fibers (P1-630PM-FC-2 for 709 nm and P1-780PM-FC-2 for 888 nm, Thorlabs), and were superimposed on a long-wavelength-pass filter (LP02-785RU-25, Semrock) in the head. The overlapped beams were radiated onto the sample with a pair of galvanometer mirrors (GVS002, Thorlabs). The backscattered CARS was separated from the excitation beams through optical filters and was detected with the photomultiplier tube (PMT) under the same bias voltage as in the CARS microscopy described above. The powers of the two excitation beams on the sample plane were 101 mW for 709 nm and 55 mW for 888 nm. The peak irradiances of $\omega _p$ and $\omega _s$ were 2.9 and 0.9 $\mathrm {GW/cm^2}$, respectively. These were lower than the reported damage threshold of 20 $\mathrm{GW/cm^2}$^42^. We observed 100 nerve images (field of view of $262\, \upmu \mathrm {m}$, $500 \times 500$ pixels, imaging rate: 37.5 images/min, exposure time: 1.6 s) at one position with the CARS endoscope, and prepared a total of 500 nerve images at 5 positions. An average image of 100 images at one position was used as a ground truth image. In the endoscopy, an objective (OL2) and a photodiode (PD) were used to obtain the transmittance images to confirm and adjust the sample positions.

### Deep learning with three models

We applied three denoising models, DN, N2N and W5, to remove noise from CARS images of nerves. The architectures are shown in Fig. 5a–c. DN and W5 are composed of skip connections, convolution and activation (ReLU: Rectified Linear Unit^43^) layers. N2N is based on Unet^22^ and is composed of skip connections, convolution, activation (leaky ReLU^44^ with $\alpha = 0.1$), max-pooling and up-convolution layers. N2N is different from DN and W5 in that ground truth images are not needed.

The hyperparameters, that is, the number (F) and size (K) of filters in the convolution layers and the number (L) of convolution layers, characterize the model and determine its performance. The hyperparameters were examined by using a grid search, as shown in Table 4. In N2N, F shows the number of filters in the convolution layers of the encoder part. F in the decoder part except for the last three convolution layers was 2F. The number of the filter of the last convolution layers in W5 and N2N was fixed to 1. The number of the filter of the second and third convolution layers from the last in N2N was 32 and 64 for all hyperparameters, respectively. The size of the filters in the convolution layers in N2N was fixed to $3\times 3$ because the performance is low when K of N2N is larger than $3\times 3$. We trained models with every combination of hyperparameters and chose the highest performance parameter for validation datasets. The hyperparameters (F, K, L) were (32, 5, 10) in DN, (48, 3, 18) in N2N, and (16, 3, 8) in W5.

We used a mini-batch size of 32 and MSE as a loss function, which measured the difference between a denoised output image and a related ground truth image. The weights of the convolution layers were optimized with the Adam optimizer^45^ with $\beta _1 = 0.9$, $\beta _2 = 0.999$, $\epsilon = 10^{-8}$ and a learning rate of $10^{-3}$. CARS microscopy images were divided into 81 images for training, five images for validation and five images for testing. CARS endoscopy images were divided into three images for training, one image for validation and one image for testing. In the case of the CARS endoscope, considering the small number of data, 5-fold nested cross-validation was adopted. All 20 possible distribution patterns were considered when the number of training images, validation images, and test images was 3:1:1, and the average evaluation index was calculated using a total of 20 models obtained. As data augmentation, random shuffle and random cropping were adopted. The flip and rotation were not utilized to avoid the influence of electronic temporal response associated with raster scanning. Except for the N2N case, random cropping cut out 50 images with $100\times 100$ pixels from an original image ($500\times 500$ pixels). In N2N, the size of the cropped images was $128\times 128$ pixels. The dataset for CARS microscopy included 4050 images for training and 250 images for validation, and that for CARS endoscopy included 150 images for training and 50 images for validation. We implemented the networks in Pytorch^46^ and processed them on a desktop computer equipped with a Core i7-8900K CPU (Intel) and an RTX 2080Ti GPU card (NVIDIA).

The accuracy of the models was assessed by using the PSNR and SSIM. The Friedman test and a two-tailed paired t-test were used for statistical comparisons. The Friedman test was utilized to determine the difference in denoising performance for each metric. The null hypothesis is that the mean ranks of the denoising performance are equal, and the alternative hypothesis is that at least one pair of mean metric is significantly different. The Friedman test was applied to compare 4 models in Table 1 and 13 models in Table 2. We utilized multiple tests for comparing the performance of denoising. The Bonferroni-Holm method was used to correct for multiple testing. The accuracy between models was evaluated by a two-tailed paired t-test ($\alpha = 0.05$).

## Supplementary information

Supplementary Information.
