# Author Correction: Improvement of nerve imaging speed with coherent anti-Stokes Raman scattering rigid endoscope using deep-learning noise reduction

**DOI:** 10.1038/s41598-020-80630-5

**Published:** 2021-01-21

**Authors:** Naoki Yamato, Hirohiko Niioka, Jun Miyake, Mamoru Hashimoto

**Affiliations:** 1 Graduate School, Faculty of Information Science and Technology, Hokkaido University, Kita 14 Nishi 9 Kitaku, Sapporo, 060‑0814 Japan; 2 Institute for Datability Science, Osaka University, 2‑8 Yamadaoka, Suita, 565‑0871 Japan; 3 Hitz Research Alliance Laboratory, Graduate School of Engineering, Osaka University, 2‑8 Yamadaoka, Suita, 565‑0871 Japan

Correction to: *Scientific Reports* 10.1038/s41598-020-72241-x, published online 16 September 2020.

In the original version of this Article, Hirohiko Niioka was omitted as a corresponding author. Correspondence and request for materials should also be addressed to niioka@ids.osaka-u.ac.jp. This error has now been corrected in the HTML and PDF versions of the Article.

