# Supplementary material for: Improvement of nerve imaging speed with coherent anti-Stokes Raman scattering rigid endoscope using deep-learning noise reduction
Source: Sci Rep. 2020 Sep 16;10:15212. doi: 10.1038/s41598-020-72241-x (PMC7495488; doi:10.1038/s41598-020-72241-x)
Supplement: Supplementary file 1 — Supplementary Information. [file 41598_2020_72241_MOESM1_ESM.pdf]

Improvement of nerve imaging speed with coherent anti-Stokes Raman scattering rigid endoscope using deep-learning noise reduction

Naoki Yamato, Hirohiko Niioka, Jun Miyake and Mamoru Hashimoto

Supplementary information

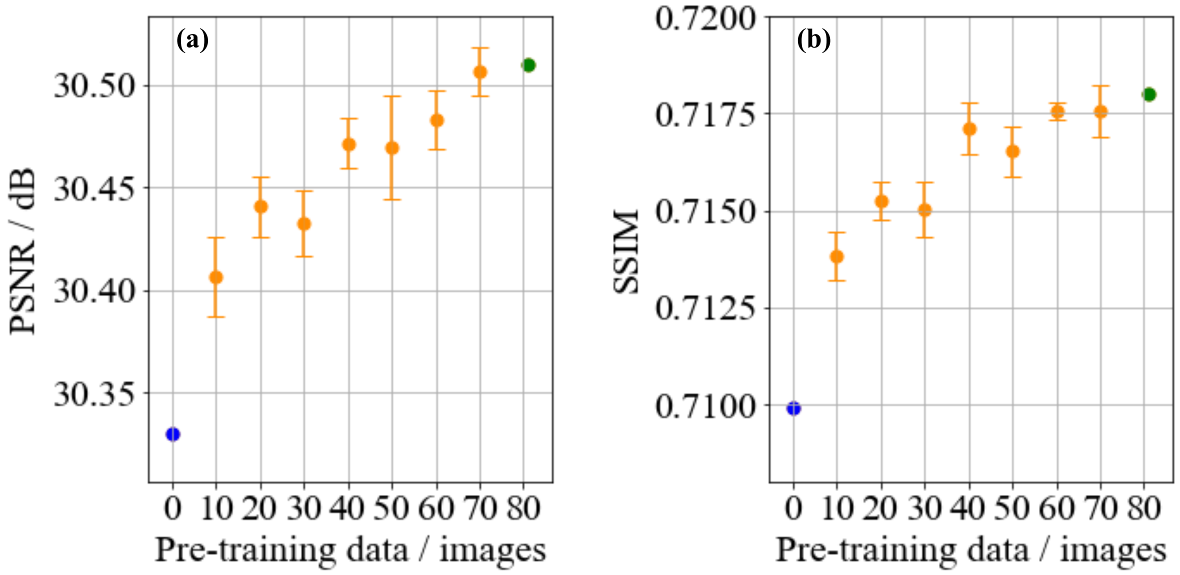

Figure S1 Results of N2N with fine-tuning for the amount of pre-training images of CARS microscopy. The metrics for test images of CARS endoscopy at 12.5 images/min (exposure time of 4.8 seconds) are shown. The blue plots are the results of N2N with endoscopy, the green plots are N2N with fine-tuning shown in Table 2. The amount of pre-training images of CARS microscopy was changed from 10 to 70 (step: 10). The pre-training images at each amount were randomly selected from 81 images 5 times. Each plot from 10 to 70 shows the average and standard deviation of 5 results. As the pre-training images increase, both evaluation metrics are improved.
